# Effect of free running wheel exercise on renal expression of parathyroid hormone receptor type 1 in spontaneously hypertensive rats

**DOI:** 10.14814/phy2.13842

**Published:** 2018-09-10

**Authors:** Katja Braun, Felix Atmanspacher, Rolf Schreckenberg, Ivica Grgic, Klaus‐Dieter Schlüter

**Affiliations:** ^1^ Physiologisches Institut Justus‐Liebig‐Universität Gießen Gießen Germany; ^2^ Klinik für Innere Medizin und Nephrologie Philipps‐Universität Marburg Marburg Germany

**Keywords:** Essential Hypertension, parathyroid hormone‐related peptide, physical Activity

## Abstract

An active lifestyle is generally recommended for hypertensive patients to prevent subsequent end‐organ damage. However, experimental data on long‐term effects of exercise on hypertension are insufficient and underlying mechanisms are not well understood. This study was aimed to investigate the effect of exercise on renal expression of parathyroid hormone‐related protein (PTHrP) and parathyroid hormone receptor type 1 (PTHR1) in spontaneously hypertensive rats (SHR). Twenty‐four rats started free running wheel exercise at the age of 1.5 months (pre‐hypertensive state) and proceeded for 1.5, 3.0, 6.0, and 10.0 months. Thirty rats kept under standard housing conditions were used as sedentary controls. Kidney function was assessed by measuring plasma creatinine levels and urine albumin‐to‐creatinine ratios. Renal expression of PTHrP and PTHR1 was analyzed by qRT‐PCR and western blot. Renal expression of PTHR1 was markedly increased between the 6th and 10th months in sedentary rats and this increase was significantly lower in SHRs with high physical activity on mRNA (−30%) and protein level (−27%). At the same time, urine albumin‐to‐creatinine ratio increased (from 65 to 231 mg/g) but somehow lower in exercise performing SHRs (48–196 mg/g). Our data suggest that enhanced exercise, stimulated by allocation of a free running wheel, is associated with lower PTHR1 expression in SHRs and this may contribute to preserved kidney function.

## Introduction

Hypertension is a major cardiovascular risk factor (Stokes et al. 1989). There are multiple mechanisms by which hypertension causes life‐time limiting mal‐adaptations such as cardiac hypertrophy or chronic kidney disease (McMaster et al. 2015). The kidney is involved in the regulation of blood pressure but also highly susceptible to hypertension‐related end‐organ damage (Morgan 2003). Spontaneously hypertensive rats (SHR) are a well‐established model of essential hypertension and kidney disease (Hultström 2012). They develop spontaneously a hypertensive phenotype at the age of 8–10 weeks that can be tolerated by the animals for various months. Finally, these rats develop severe cardiac hypertrophy with extensive cardiac fibrosis and diastolic heart failure but also kidney disease (Hultström 2012; Maskali et al. 2014). This can be prevented by inhibition of the renin‐angiotensin‐system (RAS) that reduces the blood pressure and reverses adverse remodeling of the heart (Linz et al. 1999, 2006). It is generally assumed that hyperactivity of the RAS contributes to disease progression in SHR.

An active lifestyle is usually recommended to protect the heart and kidney from end‐organ damage in the presence of hypertension (Sharman et al. 2015). Some experimental studies suggest major benefits with exercise, reporting a reduction in blood pressure, cardiac hypertrophy, and heart function although these effects could not be confirmed in older rats or if the follow‐up time is prolonged (Schlüter et al. 2010). Using a free running wheel model for rodents we previously found an inverse correlation between the dose of exercise and survival (da Costa Rebelo et al. 2012). Exercise induced excessive cardiac hypertrophy and cardiac fibrosis in female SHR (da Costa Rebelo et al. 2012; Schreckenberg et al. 2017). Our data suggested that high physical activity may not have solely protective effects on the heart in the setting of hypertension but it remains elusive how exercise affects the kidney.

Here we addressed the question whether high physical activity, performed as free running wheel exercise, has an impact on renal expression of the parathyroid hormone receptor type 1 (PTHR1, also known as PTH/PTHrP receptor) and its corresponding agonist parathyroid hormone‐related protein (PTHrP) in SHRs. PTHrP and PTHR1 are expressed in various parts of the kidney such as glomeruli, proximal tubuli, cortical thick ascending limbs, medullary thick ascending limbs, and vascular smooth muscle cells (Lee et al. 1996; Yang et al. 1997; Largo et al. 1999). Moreover, renal expression of PTHrP and PTHR1 is induced under stress conditions such as acute renal injury, diabetic kidney disease, and obstructive nephropathy (Largo et al. 1999; Romero et al. 2013). However, the expression of PTHR1 can also be downregulated in end‐stage renal failure (Kuwahara et al. 2007; Urena et al. 1994). Downregulation of PTHR1 in end‐stage renal disease may simply be a result of elevated plasma calcium under these conditions as speculated by Largo et al. (1999). Our focus was set on renal PTHR1 expression as this receptor plays different roles in normotensive rats and SHRs (Fiaschi‐Taesch et al. 1998; Massfelder et al. 2001). Whereas it dilates renal vessels in normotensive rats it has the opposite effect in hypertensive kidneys. Furthermore, SHRs display lower renal expression of PTHR1 than their normotensive counterparts (Wistar rats) (Massfelder et al. 2002; Welsch et al. 2006). Starvation simultaneously increased the expression of PTHR1 in bone and kidney (Kawane et al. 1997). Furthermore, high phosphate diet reduced renal mRNA expression of PTHR1 (Katsumata et al. 2004). These results suggest that renal expression of PTHR1 can be targeted by nonpharmacological procedures. As renal PTHR1‐dependent processes are supposed to be maladaptive in SHRs, a downregulation of PTHR1 in the kidney would properly be beneficial. We addressed the question whether physical activity affects renal expression of PTHR1 or of its endogenous agonist PTHrP because high physical activity is associated with lower risk of hypertension and subsequently hypertensive disease progression. Beneficial training effects of free running wheel exercise in these animals have been described before. These include a lower resting heart rate and improved mitochondrial function in skeletal muscles (Schreckenberg et al. 2017). However, such short‐term adaptations did not prevent heart failure in SHRs with high physical activity. In this part of our investigations we addressed the question whether prolonged high physical activity affects also kidney function in these animals, and even more specifically, PTHrP or PTHR1 expression.

## Materials and Methods

The investigations are in agreement with the “Guide for the Care and Use of Laboratory Animals” published by the US National Institute of Health (NIH Publication No. 85‐23, revised 1996). The study was approved by the local authorities (RP Gießen; V 54 – 19 c 20 15 h 01 GI 20/1 Nr. 77/2014).

### Animals and exercise model

Female SHR were randomized selected and kept either under standard conventional housing conditions (sedentary rats) or received free access to a running wheel during the whole period starting at the 6th week. Running distance, running time, and blood pressure were already reported for these animals in a former study that analyzed cardiac function and adaptations (da Costa Rebelo et al. 2012; Schreckenberg et al. 2017).

Experiments were performed on 54 SHRs subdivided into nine groups (*n* = 6 each). After 1.5, 3.0, 6.0, and 10.0 months rats were sacrificed. Sedentary controls had an additional group sacrificed after 18 months. After 6.0 and 10.0 months, blood samples were by punctuation of the *vena*. At the same time and urine samples were collected to analyze urine albumin/creatinine ratio.

### Preparation of the kidney

At the end of the experimental period rats were anaesthetized by Isofluran inhalation. After cervical dislocation kidneys were extracted, weighted, and immediately transferred to fluid nitrogen and stored until use for analysis.

### RNA isolation and real‐time RT‐PCR

Total RNA was isolated from kidney using peqGOLD TriFast (Peqlab, Biotechnologie GmbH, Germany) according to the manufacturer's protocol. To remove genomic DNA contamination, isolated RNA samples were treated with 1 U DNase/*μ*g RNA (Invitrogen, Karlsruhe, Germany) for 15 min at 37°C. One microgram of total RNA was used in a 10 *μ*L reaction to synthesize cDNA using Superscript RNase H Reverse Transcriptase (200 U/*μ*g RNA, Invitrogen, Karlsruhe, Germany) and oligo (dTs) as primers. RT reactions were performed for 50 min at 37°C. Real‐time quantitative PCR was performed using MyiQ^®^ detection system (Bio‐Rad, Munich, Germany) in combination with the iTaq Universal SYBR Green Real‐Time PCR Supermix (Bio‐Rad, Munich, Germany). The thermal cycling program consisted of initial denaturation in one cycle of 3 min at 95°C, followed by 45 cycles of 30 sec at 95°C, 30 sec at the individual annealing temperature for each primer, and 30 sec at 72°C. The sequences of the primers used in this study are indicated in Table 1. Quantification was performed as described before (Livak and Schmittgen 2001).

**Table 1:** List of primers used in this study

Gene of interest	Forward	Reverse
PTH‐1R	GTGAGGTGCAGGCAGAGATT	TCGTGTGAGACACCATTGGG
PTHrP	GCTTGGTCGCAGGCTAAAAC	TTTTGGTGTTGGGAGCAGGT
HPRT	CCAGCGTCGTGATTAGTGAT	CAAGTCTTTCAGTCCTGTCC

### Determination of plasma creatinine and urine creatinine and albumine

Creatinine concentration was analyzed in plasma samples that had been stored at −80°C. Creatinine and albumin concentrations were analyzed in urine samples that had also been stored before at −80°C. Creatinine and albumin were analyzed via standard ELISA technique as described by the manufactures manual.

### Western blot

Tissue samples were incubated in lysis buffer as described before for cardiac tissue (Schreckenberg et al. 2009). Samples (100 *μ*g) were loaded on a 12.5% SDS‐PAGE and blotted onto membranes as described before (Schreckenberg et al. 2009). Blots were incubated with a monoclonal antibody directed against PTHR1 (antibody SAB4502493, Sigma‐Aldrich, St. Louis, USA) as described before (Röthig et al. 2012) and a second antibody coupled to horseradish peroxidase (anti‐Actin [antibody A2668, Sigma‐Aldrich, St. Louis, USA]) and anti‐GAPDH (antibody 6C5 from Calbiochem EMD Chemicals, San Diego, USA). Specificity of the PTH‐1R antibody was proven by strong exposure of the samples and the absence of upcoming unspecific bands under these conditions and downregulation of the receptor in microvascular endothelial cells (see Röthig et al. 2012). Linearity of the signal was also confirmed by diluting the samples (see Fig. S1).

### Statistics

Data are expressed as indicated in the legends. Shapiro–Wilk test was used to examine whether the samples in each individual group are normally distributed. Levene's test was used to investigate whether samples from two groups have an equal variance. Subsequently, two‐side T‐Tests were performed and exact *P*‐values are given in the figures. SPS22.0 was used to calculate these data.

## Results

### Effect of exercise on kidney weight and function

**Figure 1:**
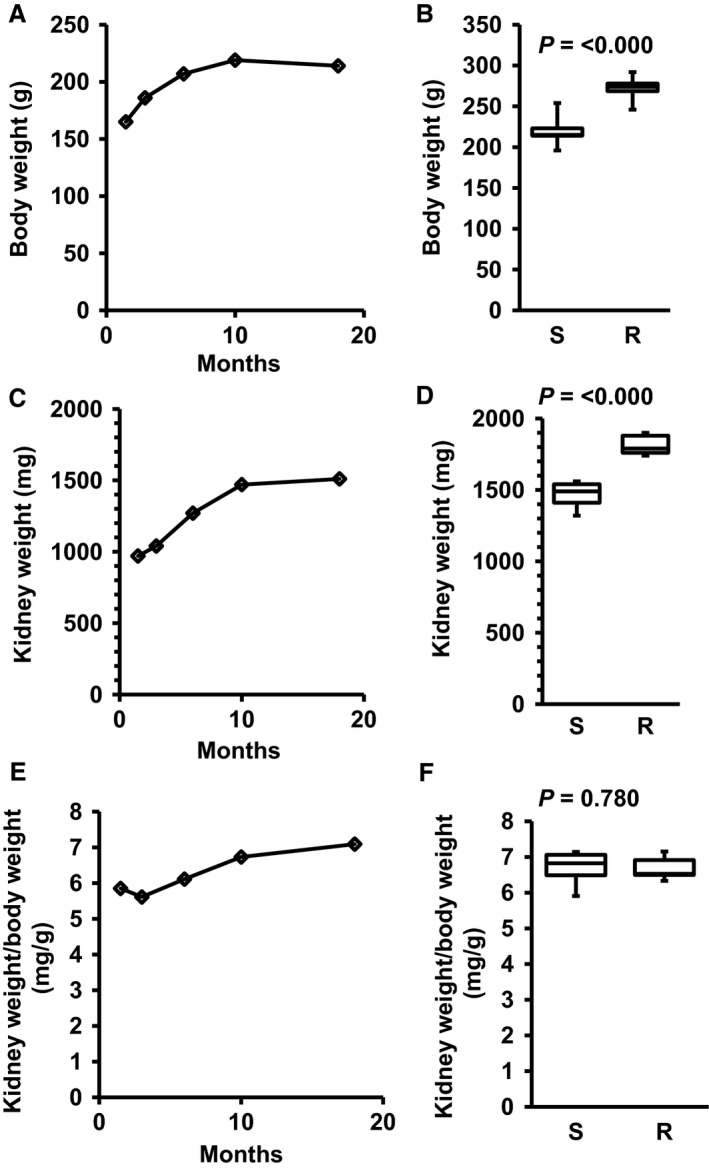
Development of body weight (A), kidney weight (C), and kidney weight to body weight (E) in sedentary SHRs (S) and the effect of free running wheel exercise (R) on these parameters (B, D, F) after 10 months. Data are means for time profiles and box plots representing the 25th, 75th quartile, and median, with whiskers representing the lowest and highest values (range). Data are from *n* = 6 rats at each time point and group. Exact *P*‐values are given.

During an 18 months’ time period, body weight initially increased during the first 10 months and remained stable thereafter (Fig. 1A). Body weights of SHRs performing free running wheel exercise were higher at all time points. The data at 10 months are given in Figure 1B. Similarly, kidney weights increased in parallel (Fig. 1C). Again, SHRs performing exercise had higher kidney weights (Fig. 1D). Kidney weights normalized to body weights slightly increased over time (Fig. 1E) but after normalization sedentary and exercise rats displayed no differences (Fig. 1F).

**Figure 2:**
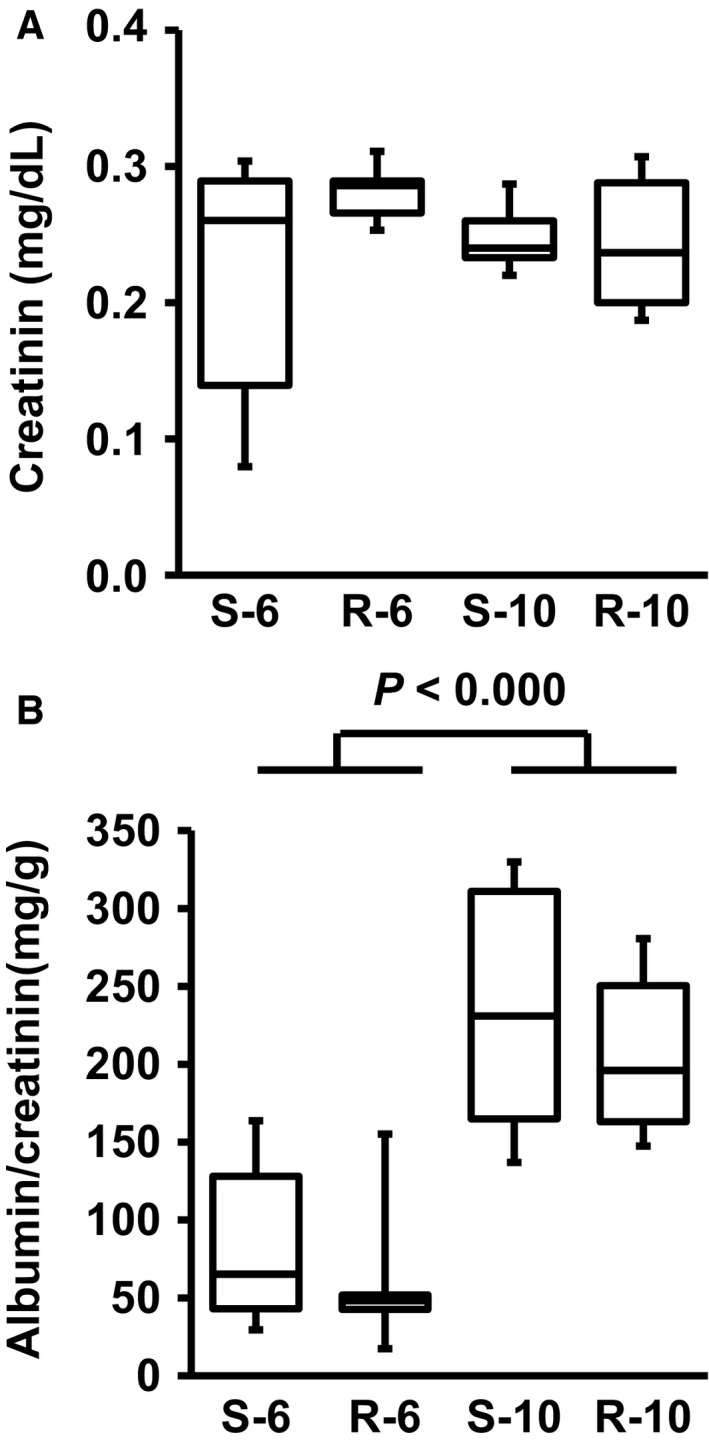
Plasma creatinine (A) and urine albumin‐to‐creatinine ratios (B) from sedentary (S) or running (R) SHRs at the age of 6 or 10 months. Data are means for time profiles and box plots representing the 25th, 75th quartile, and median, with whiskers representing the lowest and highest values (range). Data are from *n* = 6 rats at each time point and group. *P* > 0.05.

Plasma creatinine concentrations were not different between sedentary and exercise performing SHRs at 6 or 10 months (Fig. 2A). However, albumin‐to‐creatinine ratio increased significantly between 6 and 10 months of age. There was a trend to lower albumin‐to‐creatinine ratios in rats performing exercise (Fig. 2B).

### Effect of exercise on renal expression of PTH‐1R and parathyroid hormone‐related protein (PTHrP)

**Figure 3:**
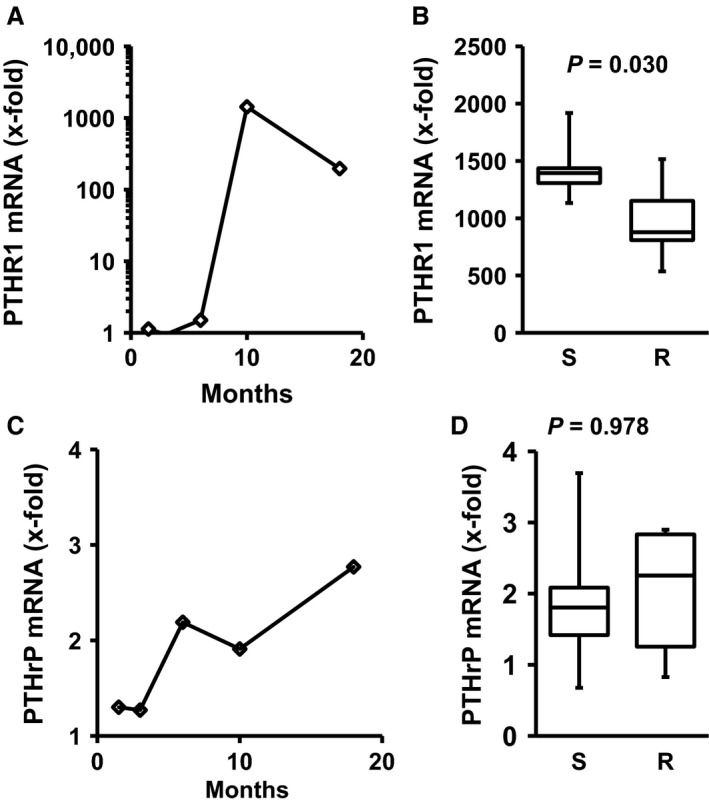
Time‐dependent renal expression of PTHR1 and PTHrP and the effect of exercise after 10 months free running wheel exercise. Data are means for time profiles and box plots representing the 25th, 75th quartile, and median, with whiskers representing the lowest and highest values (range). Data are from *n* = 6 rats at each time point and group. Exact *P*‐values are given.

The mRNA expression of the PTHR1 increased significantly within time, specifically between the 6th and 10th month (Fig. 3A). Performing free running wheel exercise significantly attenuated the increase in PTHR1 mRNA expression at that time, leading to significantly lower levels of PTHR1 mRNA after 10 months (Fig. 3B). The mRNA expression of the corresponding cytokine PTHrP also increased within time, but to a lesser extent than the corresponding receptor (Fig. 3C). Exercise did not modify the expression of PTHrP mRNA (Fig. 3D).

**Figure 4:**
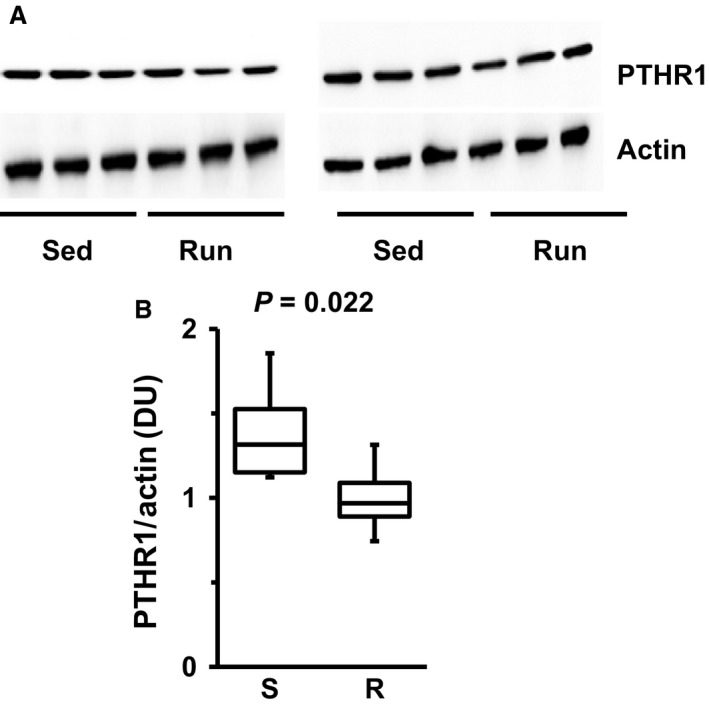
Western blots indicating renal protein expression of PTHR1 in six SHRs after 10 months of exercise. (A) Original Western Blots for PTHR1 and actin, taken as a loading control. (B) Densitometry of the blots and quantification as densitometric units (DU) of PTHR1 normalized to actin. Data are means for time profiles and box plots representing the 25th, 75th quartile, and median, with whiskers representing the lowest and highest values (range). Data are from *n* = 6 rats. Exact p‐values are given.

The data on mRNA expression suggest that exercise attenuates the induction of renal expression of PTHR1 in the kidney of SHRs. Finally, it was investigated whether this holds also on the protein level. As indicated in Figure 4, even PTHR1 protein expression was significantly downregulated by exercise in these rats.

## Discussion

The kidney is an organ required for blood pressure control but it is also susceptible to hypertension‐associated end‐organ damage resulting in glomerulosclerosis and progressive kidney failure. Hence strategies that aim at reducing elevated blood pressures are also expected to protect the kidney. An active lifestyle is recommended as first‐line “intervention” to prevent the onset of hypertension and to reduce established hypertension and its subsequent complications including end‐organ damages. Although the benefits of exercise have been well documented (Kohzuki et al. 2001; Ciampone et al. 2011; Garcia‐Pinto et al. 2011; Agarwal et al. 2012; Ito et al. 2013), experimental data suggest that there may also be an increased risk for further progression of hypertension‐associated kidney damage associated with extensive exercise (Clorius et al. 1987, 2002; Kuru et al. 2005). Moreover, recent clinical data suggest a rather complex relationship between exercise and hypertension that needs to be revisited as there may be important reasons for a more personalized approach (Allesoe et al. 2016; Williamson et al. 2016).

The data of our study suggest that exercise has a beneficial effect on the kidney in SHRs and attenuates the increased renal expression of the PTHR1 during aging. The cellular localization of PTHR1 was not further analyzed in detail in this study, but in a model of tubulointerstitial damage a similar downregulation of the receptor occurs without subsequent changes in cellular location (Largo et al. 1999). Based on these previous findings, we would not expect a cell‐specific effect. Interestingly, this effect of high physical activity on PTHR1 expression occurred at a time where increased albumin‐to‐creatinine ratios suggest the onset of renal damage (proteinuria) due to the constitutively high blood pressure in these rats. Although the low number of rats in each individual group does not allow strong correlations, it must be said that lower expression of PTHR1 was associated with lower albumin‐to‐creatinine ratios. As shown before, PTHrP contributes to proteinuria (Romero et al. 2013). Interestingly, in human tissue samples from patients with renal disease, the strongest downregulation of PTHR1 was found in those patients that had chronic glomerulonephritis with normal renal function and downregulation occurred prior to renal failure, whereas in end‐stage renal failure PTHR1 was nearly normalized again (Chen et al. 1998). Hyperactivation of PTHrP in the kidney is linked to more intensive proinflammatory effects in the kidney (Kramann and Schneider 2013). Altogether, a downregulation of PTHR1 seems to be protective is associated with a better renal function. The second important new finding of this study is the strict correlation between mRNA and protein expression in these samples. Finally, it is important to show that the expressions of the receptor but not of the agonist, PTHrP, are effected by exercise.

Little is known about the regulation of renal PTHR1 in vivo. Vitamin D, PTH, and corticosterone have been discussed but mainly excluded as potential stimuli to regulate PTHR1 expression (Turner et al. 1995). Whereas starvation increased the expression of PTHR1 in the kidney, high phosphate diet decreased the renal expression of PTHR1 (Katsumata et al. 2004). Again, a potential molecular mechanism has not been identified for either of the two mechanisms. In SHRs performing free running wheel exercise, trained SHRs had higher plasma PTH concentrations (Umemura et al. 1992). However, it is unlikely that high PTH directly reduces renal PTHR1 expression. On the other hand, it was shown that the lower expression of PTHR1 in SHRs compared to normotensive rats is caused by hyperactivation of the RAS (Welsch et al. 2006). Although angiotensin II is able to destabilize mRNAs from PTHR1, it is again unlikely that this occurs in the running rats investigated here, because there is no evidence for hyperactivation of RAS by high physical activity. Alternatively it may be assumed that an upcoming proinflammatory effect in old running rats may trigger destabilization of PTHR1 (Sipos et al. 2008). Furthermore, we have recently shown that nicotine reduces PTHR1 expression in microvascular endothelial cells by activating acetylcholine receptors (Röthig et al. 2012). Considering that high physical activity is associated with an activation of the parasympathetic nervous system as indicated by lower resting heart rate (Schreckenberg et al. 2017) it might be that acetylcholine also triggers a downregulation in the kidney of SHRs with high physical activity.

Renal PTHR1 mediate the effects of parathyroid hormone (PTH), a systemic hormone involved in calcium handling, and that of PTHrP, a locally formed cytokine (Esbrit et al. 2001). In the current study we found an age‐dependent increase of PTHR1 expression in the kidney. In normotensive rats, PTHR1 activation reduces the renovascular resistance. However, in SHR PTHR1 stimulation does not exert a similar effect but increases the proliferation of smooth muscle cells in these vessels (Massfelder et al. 2001, 2002). Therefore, we assume that the lack of an age‐dependent increase in PTHR1 in exercise performing SHRs may contribute to protective effects of exercise in hypertensive rats. Based on the studies by Massfelder et al., it is assumed that increasing the renal expression of PTHR1 in SHRs reduces the renovascular resistance leading to elevated renin levels (Massfelder et al. 2002). This would suggest that a lower expression of PTHR1 represses renin in the kidney. Indeed, we found in the exercise group a significant lower renin expression (data not shown). This may be considered as a very sensitive readout for a functional consequence of lower PTHR1 expression in this model. However, the subsequent physiological consequences are ignorable because this downregulation on the mRNA level was not accompanied by a subsequent lower renin protein expression in accordance with the still high blood pressure in the rats. Thus, although the renal expression level of PTHR1 modifies plasma renin activity as well as renin expression it does not affect blood pressure suggesting a more complex interplay between PTHrP and renin.

In contrast to the renal expression of PTHR1 that of the corresponding agonist, PTHrP, was not affected by exercise. Renal expression of PTHrP is increased under various conditions such as acute renal injury, diabetic kidney disease, and obstructive nephropathy (Largo et al. 1999; Santos et al. 2001; Izquierdo et al. 2006).It has been shown before, that angiotensin II increases the expression of PTHrP (Pirola et al. 1993). In the current study we found an age‐dependent increase of PTHrP expression. This may be linked to the activation of the RAS in SHRs. In that case, exercise has no effect on the RAS in these rats and this would be in accordance with the still increased blood pressure irrespectively of the exercise. Finally, although not specifically addressed in this study we did not find comparable changes in normotensive rats when sedentary and exercising rats were compared (data not shown). Therefore, the regulation of PTH1R shown in this study seems to be associated with hypertension.

## Conclusion

In this study we demonstrate the effect of high physical activity on renal expression of PTHR1 in SHRs and thereby suggest that this is partly involved in protective mechanisms exerted by an active lifestyle. Furthermore, our study indicates some protective effects of high physical activity on the kidney in the same animals in which cardiac function is already affected by hypertension and furthermore by exercise (this study vs. Schreckenberg et al. 2017). In summary, the kidney seems to be less sensitive to additive stress performed by long‐term exercise compared to the heart. However, as the rats start to perform exercise already in the pre‐hypertensive phase, it cannot be concluded that increasing physical activity at later time points with already existing hypertension leads to similar protection of the kidney. On the other hand, this study suggests based on an experimental study in SHRs that a more active lifestyle attenuates long‐term effects of high blood pressure in essential hypertension based on a genetic predisposition.

## Conflict of Interest

The authors of this manuscript state that they do not have any conflict of interest and nothing to disclose.

## Supporting information




**Figure S1.** Validation of Western blots indicating that the samples shown in Fig. 4 are indeed in the linear range of density. A) Original blot with PTH1R antibody and re‐probed by GAPDH. B) Quantification of the three dilution steps (1:1 shown in Fig. 4). C) Comparison between sedentary (Sed) and Running (Run) samples.
