# Supplementary material for: Effect of free running wheel exercise on renal expression of parathyroid hormone receptor type 1 in spontaneously hypertensive rats
Source: Physiol Rep. 2018 Sep 10;6(17):e13842. doi: 10.14814/phy2.13842 (PMC6129773; doi:10.14814/phy2.13842)
Supplement: Supplementary file 1 — Figure S1. Validation of Western blots indicating that the samples shown in Fig. 4 are indeed in the linear range of density. A) Original blot with PTH1R antibody and re‐probed by GAPDH. B) Quantification of the three dilution steps (1:1 shown in Fig. 4). C) Comparison between sedentary (Sed) and Running (Run) samples. [file PHY2-6-e13842-s001.pptx]

## Slide 1
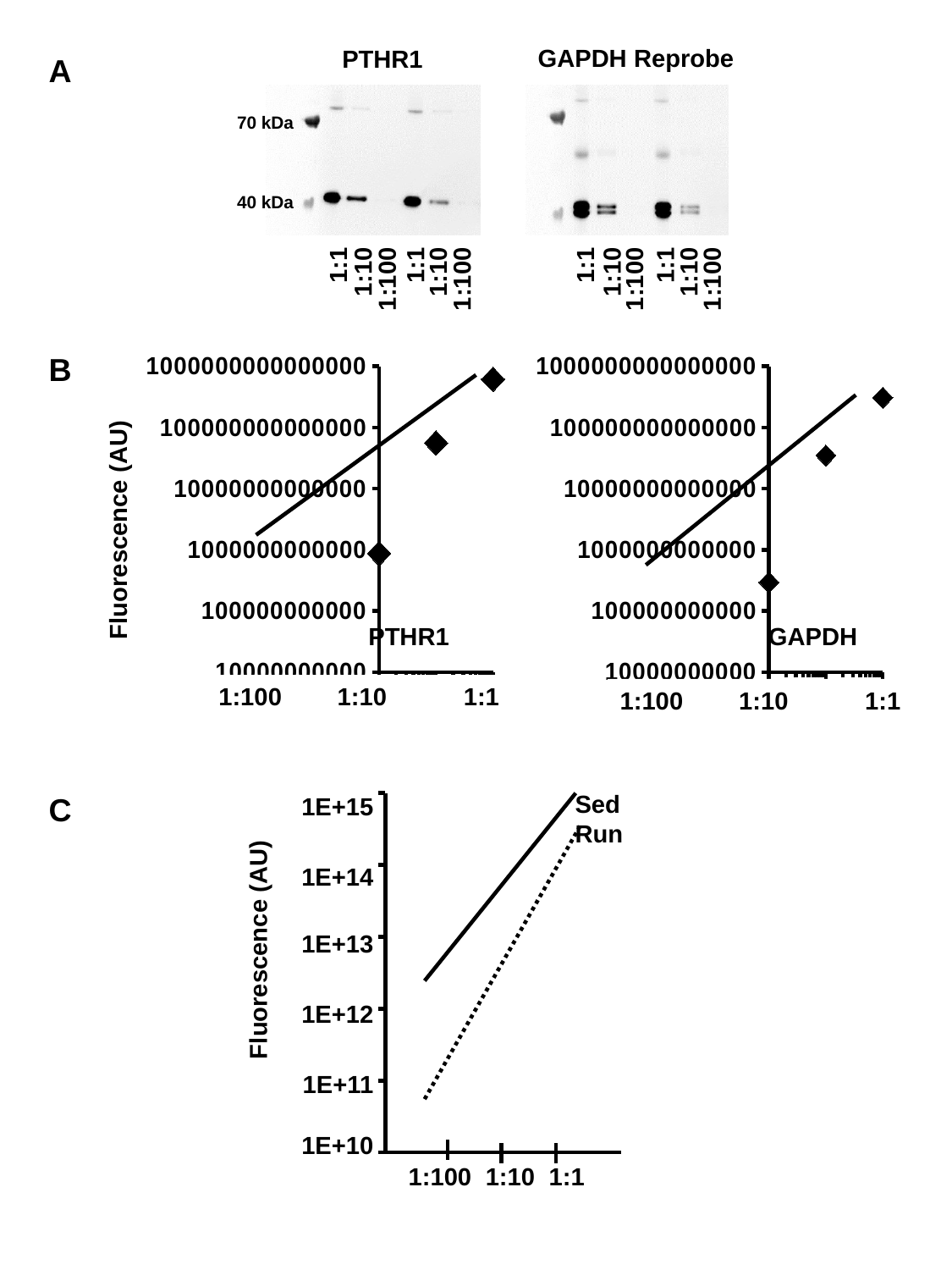

GAPDH Reprobe
PTHR1
A
70 kDa
40 kDa
1:10
1:10
1:1
1:10
1:10
1:100
1:1
1:100
1:100
1:100
1:1
1:1
B
### Chart
| Category | |
|---|---|
### Chart
| Category | |
|---|---|Fluorescence (AU)
PTHR1
GAPDH
1:100 1:10 1:1
1:100 1:10 1:1
Sed
Run
C
1E+15
### Chart
| Category | Sed | Run |
|---|---|---|1E+14
1E+13
Fluorescence (AU)
1E+12
1E+11
1E+10
1:100 1:10 1:1
