# Supplementary material for: Effect of free running wheel exercise on renal expression of parathyroid hormone receptor type 1 in spontaneously hypertensive rats
Source: Physiol Rep. 2018 Sep 10;6(17):e13842. doi: 10.14814/phy2.13842 (PMC6129773; doi:10.14814/phy2.13842)
Supplement: Supplementary file 2 [file PHY2-6-e13842-s002.docx]

**Figure S1.** Validation of Western blots indicating that the samples shown in Fig. 4 are indeed in the linear range of density. A) Original blot with PTH1R antibody and re-probed by GAPDH. B) Quantification of the three dilution steps (1:1 shown in Fig. 4). C) Comparison between sedentary (Sed) and Running (Run) samples.
